# Cognitive Sparing during the Administration of Whole Brain Radiotherapy and Prophylactic Cranial Irradiation: Current Concepts and Approaches

**DOI:** 10.1155/2010/198208

**Published:** 2010-06-27

**Authors:** James C. Marsh, Benjamin T. Gielda, Arnold M. Herskovic, Ross A. Abrams

**Affiliations:** Department of Radiation Oncology, Rush University Medical Center, Chicago, IL 60612, USA

## Abstract

Whole brain radiotherapy (WBRT) for the palliation of metastases, or as prophylaxis to prevent intracranial metastases, can be associated with subacute and late decline in memory and other cognitive functions. Moreover, these changes are often increased in both frequency and severity when cranial irradiation is combined with the use of systemic or intrathecal chemotherapy. Approaches to preventing or reducing this toxicity include the use of stereotactic radiosurgery (SRS) instead of WBRT; dose reduction for PCI; exclusion of the limbic circuit, hippocampal formation, and/or neural stem cell regions of the brain during radiotherapy; avoidance of intrathecal and/or systemic chemotherapy during radiotherapy; the use of high-dose, systemic chemotherapy in lieu of WBRT. This review discusses these concepts in detail as well as providing both neuroanatomic and radiobiologic background relevant to these issues.

## 1. Introduction

Whole brain radiation therapy (WBRT) is a mainstay of therapy for the treatment of primary and metastatic tumors involving the brain [1–3]. WBRT entails treatment of the whole intracranial compartment (brain and brainstem) down to the foramen magnum or to the bottom of either the first or second cervical vertebrae, with a uniform dose of radiation, typically administered with opposed lateral fields and blocks to protect the lenses. Multiple dose fractionation regimens have been employed for WBRT, with no one schedule having been conclusively proven better than others, although single fraction therapy (ex. 10 Gy in a single fraction) has been shown to result in greater toxicity [4]. Common utilized treatment schedules include 2.5 Gy × 14 or 15 fractions, or 3 Gy × 10 fractions. In the setting of intracranial metastases, patients with RTOG RPA class III (Table 1) disease are often managed with WBRT alone, while patients with RPA class I or II disease are frequently managed by a combination of modalities, including WBRT alone, surgical resection followed by WBRT, stereotactic radiosurgery (SRS) alone (particularly for Class I patients), or SRS in combination with WBRT [5–9]. Unfortunately, WBRT is associated with late brain toxicities, which range in severity from mild deficits in cognitive dysfunction to overt dementia in up to 11% of patients depending on the population studied, the length of follow up, and the type of chemotherapy employed [10–13]. The sequelae of treatment are even more severe in pediatric patients treated with WBRT, in whom hearing loss, severe global cognitive deficiencies, and neuro-endocrine deficits may develop [14–16]. In patients 60 years of age and older with primary CNS lymphoma, the combination of WBRT and high-dose methotrexate regimens has resulted in severe to fatal leukoencephalopathy resulting in the frequent omission of cranial radiotherapy in this context [17]. 

Prophylactic cranial irradiation (PCI) has become a standard of care for selected patients with limited and extensive stage small cell lung cancer (SCLC) who have shown benefit with systemic treatment. PCI has also been explored in the context of nonsmall cell lung cancer (NSCLC), but in this context has not been shown to improve overall survival [18–22]. A recent RTOG study (RTOG 0214) exploring the use of PCI in NSCLC was presented at the 2009 ASTRO (American Society for Therapeutic Radiology and Oncology) meeting in Chicago, IL. [23, 24] 340 patients with Stage III nonsmall cell lung cancer, who showed no evidence of tumor progression after treatment of their primary tumor, were randomized to be treated with PCI or undergo observation from 2002 to 2007. PCI resulted in a reduction in the incidence of brain metastases from 18% to 8%, but did not impact overall survival [24]. Importantly, while PCI did not significantly impact overall reported quality of life, it did result in lower rates of both immediate and delayed recall, suggested that the use of PCI impairs memory function in treated patients [24]. 

The concept underlying PCI is to eliminate microscopic deposits of metastatic tumor within the brain and/or brainstem before they become clinically manifest. Without PCI, more than 60% of small cell lung cancer patients will eventually develop clinically detectable and/or symptomatic brain metastases at some point during the course of their disease, and PCI reduces this rate to approximately 20% [25]. The treatment field for PCI is similar to WBRT in that the whole brain and brainstem down to the foramen magnum or the bottom of the first or second cervical vertebrae is treated to a uniform dose, with most patients being treated using opposed laterals with lens blocks. Multiple treatment schedules are employed, with no one schedule clearly showing superiority to others [26]. A recent prospective study found that there was no significant reduction in the number of brain metastases for 36 Gy in 18 fractions versus 25 Gy in 10 fractions, while overall survival was worse for unclear reasons in the higher dose arm [26]. The authors concluded that 25 Gy in 10 fractions should remain the standard of care in this setting [26]. In the setting of both limited and extensive stage small cell lung cancer, the use of PCI has resulted in statistically significant improvements in overall survival (OS) [27, 28]. As these patients are at risk for cognitive deficits from multiple causes such as age-related cerebral atrophy, preexisting cerebrovascular disease, anxiety, depression, and chemotherapy effects, there has been controversy regarding the extent to which PCI contributes to observed neurocognitive deficits [29]. However, with recent increases in mean overall survival and an increased number of longer-term survivors, the contribution of PCI to the development of neurocognitive deficits is becoming more clearly defined [30]. Finally, PCI for pediatric patients with high risk acute lymphocytic leukemia (ALL) is known to cause significant late sequelae and this knowledge has prompted reductions in the indications for, and doses of, PCI in this context [30–34]. 

In this article we review these issues in further detail and discuss the different methods currently being employed and explored in an effort to reduce neurocognitive toxicity.

## 2. Toxicity of Cranial Irradiation

The effects of cranial irradiation may be roughly divided into acute, subacute, and chronic [35]. Acute side effects, which occur during or within a few weeks of radiation therapy, include fatigue, alopecia, nausea, and effects related to exacerbation of baseline cerebral edema such as headache, nausea, focal deficits, and when severe changes in mental status. Subacute symptoms (those occurring after the completion of radiotherapy but within three months of the end of treatment) are relatively rare and limited primarily to the somnolence syndrome and, less frequently, early onset leukoencephalopathy. The pathophysiology of the somnolence syndrome is probably related to transient demyelination of cerebral white matter (analogous to Lhermitte's syndrome after spinal irradiation). Leukoencephalopathy, on the other hand, is believed to represent a more severe manifestation of demyelination and may be fatal. These white matter changes may be more prominent in older patients with vascular risk factors, and evidence of this damage can be identified before other gross changes are evident on MRI by early changes in fractional anisotropy (FA) as identified on diffusion tensor imaging (DTI) after the delivery of PCI [36]. Similar changes in FA on DTI can be seen in pediatric patients who have been treated with radiotherapy for medulloblastoma, with one recent study showing a mean reduction in FA of 16.5% in treated patients versus controls [37]. These reductions in FA were found to correlate with a younger age at the time of treatment and declines in school performance [37]. Late side effects, which occur six months or later after radiation therapy, include overt radionecrosis of the brain (with areas of focal coagulative necrosis) and progressive microvascular or vascular occlusion with a subsequent increased risk of stroke. Rarely this may mimic Moyamoya syndrome as seen in other contexts not involving radiotherapy or malignancy [38–41]. 

 Various systems have been developed to describe these effects, including (among others) the NCI Common Toxicity Criteria Version 2.0 (available at http://ctep.cancer.gov) and the RTOG/EORTC LENT-SOMA systems [42]. These scoring systems' definitions of neurotoxicity are shown in Tables 2 and 3. However, more subtle deficits in cognitive function are not accounted for in these systems, nor are all of the neuroendocrinologic sequelae of therapy.

Late toxicities in the brain are highly feared sequelae of cranial irradiation in both adults and children because they are highly debilitating and irreversible. The axonal tracts that connect the cerebral cortex to the subcortical gangliae, spinal cord, and brain stem nuclei do so in series, such that damage to any part of the sequence adversely affects [43]. The generally accepted TD5/5 and TD50/5 (radiation doses which, when delivered to a given type of tissue in a typical patient population, will result in a 5% or 50% rate of Grade 3 or higher toxicity at a time point 5 years removed from the radiation exposure, resp.) after treating the whole brain with standard fractionation are 45 Gy and 60 Gy, respectively, and for partial brain radiation exposure are 60 Gy and 75 Gy [44]. Due to the slow rate of cell turnover for neuronal and glial elements, the brain represents a late responding tissue, with an accepted *α*/*β* ratio of about 2 Gy [45]. This suggests that treatment of the brain with smaller daily or fractional radiation doses might reduce risk of late sequelae. However, the use of smaller radiation fractions also necessitates the delivery of a higher total dose of radiation to achieve the same degree of tumor control [43].

Various treatment schedules have been developed for the administration of WBRT in the setting of brain metastases [46]. One commonly utilized schedule is 30 Gy in 10 fractions, which (assuming an alpha/beta ratio of 2 Gy for late neurologic sequelae) correlates with a biologically equivalent dose (BED) of 75 Gy_2_, theoretically below the TD5/5 of whole brain (45 Gy by standard fractionation at 2 Gy per fraction, which corresponds to a BED of 100 Gy_2_). Other commonly used schedules include 2.5 Gy × 14 or 15 fractions, which result in BED values of 78.75 Gy_2_ and 84.4 Gy_2_, respectively, again below the accepted TD5/5 for whole brain radiation exposure. Thus, all of these treatment schedules should result in rates of late neurologic sequelae that are significantly less than 5%. However, the NCI and EORTC/RTOG toxicity scoring systems (Tables 2-3) do not include the readily clinically identified changes in cognition and behavior that are well documented after these therapies [10–16].

The most frequently described adverse effects in adults treated with WBRT include problems with the consolidation of new memory, poor attention span/concentration, visual-spatial difficulties, difficulty with executive planning, and poor fine motor control [10]. A recently published study by Welzel et al. prospectively assessed cognitive function in patients being treated with either WBRT (40 Gy in 20 fractions or PCI (36 Gy in 18 fractions) at baseline, after 1–3 fractions, after the last fraction, and at 6–8 weeks after the completion of radiation therapy [13]. These authors found that acute declines in verbal memory were seen with WBRT (but not PCI) patients after 1–3 fractions and at the completion of treatment, while subacute declines in verbal memory were seen in both WBRT and PCI patients 6–8 weeks after the completion of treatment [13]. On multivariate analysis, they found that these deficits persisted even after accounting for the use of chemotherapy, KPS score, and the presence of depression and/or anxiety. They found no significant declines in visual memory or attention span [13]. 

In some cases, late neurological deficits can be severe enough to cause overt dementia, wherein the patient's global level of functioning is severely impaired and the patient is not aware of these changes. This is in contrast to more subtle cognitive deficits that are commonly seen and of which the patient is typically well aware. The incidence of dementia after cranial irradiation has been reported to be as high as 11% in patients with long-term followup [11], and these long-term sequelae have been shown to correlate with and precede decline in patient-reported quality of life (QOL), [12, 13]. 

PCI has variably been described as having no effect on cognitive function, adversely affecting cognitive function, and even initially improving cognitive function in adult patients [13, 47–51]. As reported by Welzel et al., PCI patients appear to start out with lower baseline cognitive functioning scores than the WBRT patients, have a transient improvement in simple reaction time (the ability to respond to an acute stimulus) during and at the end of radiation therapy, and subsequently have a decline in verbal memory 6–8 weeks after completing therapy [13]. A report recently released by the EORTC of patient-reported quality of life scores after PCI showed a significant decline in QOL up to 3 months after the completion of treatment, although the most frequently reported complaints were for alopecia and fatigue and the global QOL scores were less adversely affected [30]. For individual patients demonstrating brain metastases following PCI, it can be difficult to determine the relative contribution of the recurrence and the PCI when neurocognitive decline is identified [52]. Tumor progression may particularly contribute to declines in cognitive function in patients with significant peritumoral edema [53]. Finally, small cell lung cancer may, even in the absence of overt intracranial metastatic involvement, adversely affect cognitive function by mechanisms that are not clearly understood, possibly paraneoplastic [54]. 

Exposure of cerebral vasculature, particularly small arteries and arterioles, is known to cause the late development of hyaline-type arteriosclerosis with a subsequent increased risk of ischemic stroke [38]. In very young children with significant exposures to the anterior Circle of Willis region, changes can be seen which include bilateral carotid occlusion with the subsequent development of transdural anastamoses and a “net” or “cloud” of small collateral vessels; these changes collectively are known as moyamoya syndrome [38–41]. The moyamoya changes seen after cranial irradiation are essentially identical to those seen in primary moyamoya (Nishimoto's disease) and result in similar clinical manifestations such as cerebral ischemic strokes, recurrent transient ischemic attacks (TIAs), motor deficits, sensory deficits, global cognitive dysfunction, convulsions, and/or migraine-like headaches [38]. 

The significant late sequelae associated with cranial RT have stimulated interest in finding ways to avoid this toxicity without sacrificing clinical outcomes for this effective and widely available therapy.

## 3. WBRT and PCI in the Pediatric Population

WBRT is a standard part of the treatment approach for primary CNS pediatric tumors that have a propensity for dissemination along the neuraxis, including anaplastic ependymoma, medulloblastoma, ependymoblastoma, pineoblastoma, atypical rhabdoid/teratoid tumor, nonseminomatous germ cell tumor, and choroid plexus tumors. WBRT in this setting may or may not be combined with spinal irradiation, and the doses used vary depending upon the tumor type, the age of the patient, and the clinical context [55–59]. A number of approaches have been used to minimize the late toxicity of cranial irradiation for these patients.

For standard risk medulloblastoma, the dose of cranial spinal irradiation (CSI) (including the WBRT dose) has been reduced from 36 Gy to 23.4 Gy in an effort to reduce some of the late effects of cranial irradiation [60]. For intracranial germinomas either whole-ventricular radiation therapy or chemotherapy followed by involved-field radiation therapy is now preferred over WBRT, again in an effort to spare the child the late sequelae of treatment [61, 62]. Chemotherapy without radiation therapy has been utilized in very young patients (3 years of age and less) as a primary therapy, adjuvant therapy, or “bridge” therapy to delay the use of radiation therapy for primary CNS tumors until patients are older and better able to tolerate the effects of cranial irradiation [63, 64]. Chemotherapy alone or as adjuvant therapy has also been used as a treatment modality for intracranial germinomas and nongerminomatous germ cell tumors, but with unacceptably high failure rates [65].

For pediatric patients with acute lymphoblastic leukemia (ALL) and acute myelogenous leukemia (AML), WBRT is utilized as an effective therapy for patients who present with overt CNS involvement and those who relapse in the CNS [66–69]. However, given the known late effects of cranial irradiation in the pediatric population, a number of groups and institutions have developed protocols which exclude WBRT even in patients with overt CNS involvement or who relapse in the brain after initial treatment [70–72]. 

PCI is currently employed as part of standard therapy for 2–20% of patients with ALL who have no overt CNS involvement but have a number of other high risk features (age >9 years old or <1 year old, T cell phenotype, WBC greater than 50,000 or 100,000, extramedullary disease, presence of Philadelphia chromosome, and poor response to induction therapy) [66, 67]. The dose of PCI has systemically been reduced from 24 Gy to 18 Gy, and some protocols now employ doses as low as 12 Gy [73–75]. However, even at a dose of 18 Gy, there is evidence of late cognitive and neuron-endocrinologic sequelae in these patients, even when treatment is delivered on a hyperfractionated schedule of 0.9 Gy twice daily [16, 70–74]. 

St. Jude Children's Hospital has extensively studied the cognitive and other late neurologic side effects of cranial irradiation in children [15, 34, 76–78]. In one study, they found that the patient's age and the percent volume of supratentorial brain irradiated to varying dose levels (0–20 Gy, 20–40 Gy, 40–65 Gy) correlated with IQ level after cranial irradiation, with younger age at the time of treatment and the treatment of larger percent volumes of supratentorial brain to higher doses correlating significantly with declines in IQ after treatment [76]. In another study evaluating the feasibility of field reduction after resection of infratentorial ependymomas, they tested neurocognitive function at baseline and at varying time points after cranial radiation and found that patients treated with fields encompassing the tumor bed/tumor and 1 cm margin (as opposed to a typical larger field) had no detectable neurocognitive deficits after treatment, suggesting that sparing the cochlea (to preserve hearing) and avoiding irradiation of the supratentorial brain minimized the risk of late neurocognitive sequelae [77]. After partitioning the brain into 5 compartments (total brain, supratentorial brain, infratentorial brain, right temporal lobe, and left temporal lobe), they found that irradiation of the supratentorial compartment and temporal lobes resulted in significant declines in IQ regardless of dose level, with each Gy of exposure having a similar impact on declines in IQ [34]. The cognitive deficits seen after cranial irradiation seem to be due to an inability to develop new skills and to process new information, rather than a loss of previously acquired skills and information [15]. The factors that seem to correlate most strongly with cognitive decline after cranial irradiation are a younger age at the time of treatment, longer time interval since treatment, female sex, presence of hydrocephalus, higher volume of supratentorial brain irradiated, and higher radiation dose to the supratentorial brain [78]. 

Hearing loss also contributes to the learning difficulties these pediatric patients face after cranial irradiation, and can result from irradiation of the cochlea/inner ear and/or the use of ototoxic drugs such as platinum agents [75]. One of the goals of field reduction in the treatment of infratentorial pediatric brain tumors is to minimize cochlear irradiation. For example, in the context of craniospinal irradiation for the treatment of medulloblastoma, the boost field has been systematically reduced from treatment of the whole posterior fossa, to treatment of the tumor resection bed with a 2 cm margin, to recent efforts at treating the tumor resection bed with even smaller margins [14, 75, 79–81]. IMRT and proton therapy have also been utilized in the treatment of pediatric CNS tumors with the goal of reducing cochlear dose and dose to the brainstem and other critical local structures [82–85].

Thus, in the pediatric population, approaches to reducing the late neurotoxicity, endocrinopathies, and ototoxicity associated with cranial irradiation have included avoidance of cranial irradiation altogether, dose reduction, field size reduction, use of IMRT, and use of proton therapy. The growing trend in recent trials, as exemplified by the recently published Total Therapy XV study from St. Jude Children's hospital, has been to avoid cranial irradiation altogether through the use of risk-adapted intrathecal and systemic chemotherapy regimens [86].

## 4. Omission of WBRT in Primary CNS Lymphoma

The treatment of primary CNS lymphoma has evolved over the years, with earlier trials utilizing whole brain radiation therapy (WBRT) alone, and subsequent trials using induction chemotherapy followed by WBRT (with or without more chemotherapy after radiation therapy), or chemotherapy alone [17, 87–101]. RTOG 83-15 was a phase II trial which treated patients with WBRT to a dose of 40 Gy in 20 fractions, followed by a sequential boost to the patient's gross disease of 20 Gy in 10 fractions [87]. This trial resulted in a median OS of only 12.2 months and a 2-year OS of 28% [87]. Schultz et al., in a subsequent phase I/II trial (RTOG 88-06), treated patients with 2 cycles of induction CHOD (cyclophosphamide, doxorubicin, vincristine, and dexamethasone) followed by WBRT to a dose of 41.4 Gy in 23 fractions and a sequential cone down boost to the patient's gross disease of 18 Gy in 10 fractions (total 59.4 Gy) [90]. This trial produced a median OS of 16.1 months and a 2-year OS of 42%, slightly better than the results found in 83-15, but on direct comparison the difference was not found to be statistically significant, and the authors concluded that induction chemotherapy did not improve survival versus radiotherapy alone [87, 88]. Of note, both 83-15 and 88-06 found that OS was significantly improved in patients less than 60 years old [87, 88]. 

DeAngelis et al. in RTOG 93-10 treated patients with five cycles of methotrexate-based chemotherapy (IV methotrexate 2.5 g/m^2^, vincristine, procarbazine, and IT methotrexate 12 g), followed by WBRT to a dose of 45 Gy in 25 fractions and then high dose cytarabine as consolidation therapy [91]. They found a median OS of 36.9 months and a median progression-free survival (PFS) of 24 months, significantly better than the results seen in 83-15 and 88-06 [91]. As in the two prior trials, this trial found that patients younger than age 60 had a significantly better median OS (50.4 months) than patients aged 60 years or older (21.8 months) [91]. Unfortunately, they also found that 15% of the patients (12 total patients) experienced severe delayed neurotoxicity, particularly diffuse leukoencephalopathy [91]. 8 of these 12 patients died as a result of their leukoencephalopathy [91]. 

The trial was amended to allow a lower dose of hyperfractionated WBRT (36 Gy in 30 fractions, two fractions per day) given over 3 weeks for patients with a complete response (CR) to induction chemotherapy, in an effort to reduce the morbidity of treatment without compromising outcomes [92]. Unfortunately, neurocognitive outcomes as assessed by minimental status examination (MMSE) showed no significant improvement with this hyperfractionated WBRT versus standard fractionated WBRT, with 10% of the hyperfractionated patients experiencing grade 5 neurotoxicity by 4 years after treatment [92]. Also, hyperfractionation did not improve OS or PFS [92]. 

Investigators at MSKCC (Memorial Sloan-Kettering Cancer Center) have published a retrospective review of 185 patients treated with high-dose chemotherapy and WBRT and found a 24% rate of significant neurotoxicity by 5 years after the completion of treatment [98]. In a separate report, the same group reported on a series of 5 patients (median age 74 years old) who died of treatment-induced diffuse leukoencephalopathy and found that significant clinical signs of neurotoxicity could be identified as early as 1 month after the completion of therapy, suggesting that this potentially lethal consequence of treatment is not always a delayed phenomenon, but one which could be seen very early in some patients [99]. The rate of significant late neurotoxicity with combined modality therapy seems to be age related, with patients aged 60 years and older having rates of anywhere from 10% to 83% in various reports [17, 88–93]. These patients are known to have a poorer outcome than younger patients independent of the use of combined modality therapy and subsequent neurotoxicity, and age greater than 60 years old is considered a poor prognostic factor using scoring systems from MSKCC and the International Extranodal Lymphoma Study Group [100, 101]. Delayed neurotoxicity is the leading cause of morbidity after treatment and is often fatal [17, 98, 99]. Because of this high rate of toxicity, a number of groups have begun treating primary CNS lymphoma patients with chemotherapy alone, reserving radiotherapy for treatment failures [17, 97, 100, 101]. These studies have variably reported high rates of failure in younger patients (especially less than 60 years old) in some series, but survival rates in older patients are similar or superior to the results seen with combined modality therapy [17, 94–97]. This has led some investigators to conclude that combined modality therapy should be reserved for patients younger than age 60, while in older patients it should be reserved for salvage [17, 94, 96]. 

Thus, because of the high risk of delayed neurotoxicity after combined modality therapy for primary CNS lymphoma, particularly in the elderly, WBRT is increasingly being used as salvage therapy alone rather than as a component of initial therapy despite its proven efficacy [17, 91, 98, 99].

## 5. SRS as Monotherapy for Brain Metastases

Stereotactic radiosurgery (SRS) is a technique by which a single large fraction of ionizing radiation is delivered with submillimeter accuracy to a small treatment volume, most of which is tumor. Initially restricted to patients with a solitary brain metastasis, SRS has now been applied in the setting of multiple brain metastases, and as a single modality [102–113]. Because of the steep dose gradients achieved using SRS, it has been proposed as a means by which to minimize the radiation dose to normal brain, hopefully translating into an improvement in cognitive sparing. Many authors have reported local control and survival outcomes after using SRS with or without WBRT [8, 9, 102, 104, 108–110, 112, 113]. WBRT consistently improves local control and decreases distant intracranial failures, but the addition of WBRT has had an inconsistent impact on survival [8, 9, 102, 104, 107–113]. Still, it has been increasingly noted that the outcomes of survival and local control do not adequately describe the relevant outcomes in the brain metastases population; neurocognitive function (NCF) and quality of life (QOL), which has been shown to be tightly linked to NCF, are also critical endpoints which may be linked to factors other than the use of radiotherapy, such as control of progression within the CNS, use of chemotherapy, or use of antiepileptic medications [102, 103, 114–118]. In particular, some studies have found that progression of disease within the CNS is a stronger predictor of poor QOL and NCF than the toxicity of therapy, including radiotherapy, and that control of CNS disease may actually improve these outcomes [114].

Chang et al. recently published the results of a randomized controlled trial in which patients with 1–3 brain metastases were treated with SRS alone or combined with a course of WBRT (37.5 Gy in 15 fractions of 2.5 Gy each) [113]. The primary endpoint of this study was neurocognitive function as assessed by the HVLT-R (Hopkins Verbal Learning Test-Revised) at 4 months following the completion of therapy; secondary endpoints included control within the CNS and overall survival [113]. The trial was stopped after 58 patients had been enrolled due to early stopping rules because of a significant decline in memory function at 4 months following therapy in the SRS + WBRT arm of the study; no significant difference was noted in overall survival at 4 months, but the rate of intracranial failure was higher at 1 year in the SRS alone arm (73% for SRS alone versus 27% for SRS + WBRT) [113]. The authors of this study concluded that patients with 1–3 brain metastases should be managed initially with SRS alone followed by close observation [113].

Longitudinal data tracking the NCF of patients receiving WBRT, SRS, or both are sparse. Chang et al. prospectively assessed 15 patients with 1–3 metastases receiving treatment with SRS alone [103]. A comprehensive battery of tests evaluating neurocognitive function (NCF) was performed on each patient evaluating attention, memory, dexterity, and executive function. 67% of patients were found to have a deficit in at least one domain prior to treatment. In accordance with the data of others, patients with larger tumor volume (>3 cm^3^) were found to have worse NCF. Immediately following SRS, all patients experienced a decline in at least one domain, but in the 5 patients who underwent long-term followup, 80% demonstrated stable/improved learning memory and 60% had stable/improved executive function and dexterity [103]. 

Kondziolka et al. compared the morbidity of SRS and WBRT from the patient's perspective via a retrospective survey in 200 consecutive patients [112]. Patients whose treatment included WBRT felt they had significantly more problems with fatigue, short-term memory, long-term memory, concentration, depression, and fatigue. Overall, SRS was thought to be a good treatment by 76% of patients, whereas only 56% of patients thought WBRT was a good treatment [112]. 

Aoyama et al. performed prospective NCF assessment within the context of a phase III trial randomizing 132 patients between SRS + WBRT and SRS alone [102, 104]. The MMSE (Mini-Mental Status exam) was used as a surrogate for NCF and was obtained prior to treatment, 1 month after treatment, and every three months thereafter if possible. 92 patients were available for follow-up MMSE, of these, 39 were abnormal (<27) at baseline. Of these 39 patients, 20 (51%) experienced an improvement in MMSE after treatment, 9 in the SRS group, and 11 in the combined modality group. Actuarial preservation of MMSE score ≥27 at 12, 24, and 36 months was 78.8%, 78.8%, and 22.5% in the SRS + WBRT group, versus 53.3%, 42.6%, and 42.6% in the SRS alone group. Deterioration was attributed to RT toxicity in 5 patients in the SRS + WBRT group, while no patients receiving SRS alone had a toxic event. Intracranial recurrence was deemed the cause of NCF decline in 3 and 11 patients in the WBRT + SRS and SRS alone groups, respectively [102]. The data of Aoyama et al., while subject to limitations, suggests that the omission of WBRT decreases intracranial control and may negatively impact NCF over the first 12–24 months. Of concern, long-term survivors in the WBRT + SRS group appear to demonstrate a continued decline in MMSE that may represent the late toxicity of WBRT, while the long-term survivors receiving SRS alone display stable MMSE [102]. These results must be interpreted with caution, however, because of the small number of patients available for followup at the late time points [102]. 

The utilization of SRS in the absence of WBRT does not appear to be a perfect solution to the problem of neurocognitive dysfunction in patients with intracranial metastases because of the known increased rates of local progression within the brain seen in patients treated with SRS alone [102–106, 108–113]. Further understanding of the complexities of neurocognitive toxicity will only be achieved when thorough NCF evaluation is a standard part of every investigation exploring therapies for brain metastases. Now that the feasibility of large-scale NCF testing during brain metastases trials has been demonstrated, the fund of knowledge will undoubtedly grow, allowing the optimization of the therapeutic index [118].

## 6. Hippocampal Sparing

**Figure 1:**
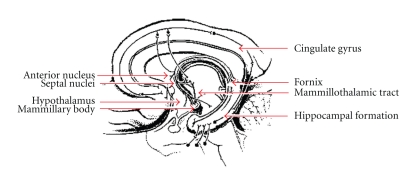
Hippocampus and Limbic Circuit (http://www.thebrain.mcgill.ca/).

Several groups have recently investigated the safety and feasibility of sparing the hippocampus while simultaneously treating the rest of the brain with radiotherapy [119–121]. The hippocampus (Figure 1) occupies the ventro-medial aspect of the temporal lobe, lying posterior to the amygdaloid complex and lateral to the temporal horn of the lateral ventricle [122]. Functionally, the hippocampus is primarily involved in the consolidation of new memories. It is composed of the dentate gyrus, which in addition to its role in memory consolidation is also important in the achieving and maintaining of “happy” states, and the cornu ammonis (CA1–CA3 regions) [123]. A specific subregion within the dentate gyrus is the subgranular zone (SGZ), which contains neural stem cells (NSC) that are involved in the repair of damage from various insults to the CNS (including radiation therapy) [124]. These cells are also important in maintaining the ability to learn throughout life. The axons that arise from hippocampal neurons become the fornix, a white matter tract that extends from the posterior aspect of the hippocampal formation and around the third ventricle (adjacent to the corpus callosum) to eventually synapse at the mammillary bodies (part of the hypothalamus), which are also involved in the consolidation of new memories [123]. 

The hippocampus is rarely involved by intracranial metastatic disease [118, 121]. Ghia et al. at the University of Wisconsin recently reviewed the records of 272 intracranial metastases and found that only 3.3% of lesions were within 5 mm of the hippocampus, while 86.4% of lesions were >15 mm from the hippocampus [119]. In a retrospective review of 697 intracranial metastases at Rush University Medical Center, only 2.29% of these lesions directly or indirectly (by secondary growth) involved the hippocampus [121]. In “oligometastatic” patients (those with 1–3 metastases only), the rate was even lower at 0.97% [121]. Because hippocampal involvement by metastatic disease is rare, and because memory loss (specifically the inability to consolidate new memories) is such a frequent and major component of late neurotoxicity from cranial irradiation, sparing of the hippocampus during the administration of WBRT or PCI should result in lower rates of memory loss [10–13, 15, 34, 63, 64, 73, 78, 119, 121] without compromise of therapeutic goal. This is particularly supported by data from St. Jude Children's Hospital, who found that the primary neurocognitive deficit noted in children exposed to cranial irradiation was the inability to form new memories, and that loss of IQ after cranial irradiation correlated with the dose delivered to the temporal lobes (the location of the hippocampi) [15, 34].

Investigators at the University of Wisconsin have shown that it is possible to selectively reduce dose to the hippocampus (maximum dose constraint 6 Gy) while treating the whole brain to a D95 of 32.25 Gy and treating metastatic lesions to much higher doses (D95 of 63 Gy for tumors 2 cm + in max. diameter, and 70.8 Gy for tumors <2 cm in max. diameter) [120]. Such significant dose reductions may be necessary to spare this structure, because the hippocampus is known to be very sensitive to radiation exposure, particularly the CA1 and subgranular zone (SGZ) regions [125–128]. Apoptosis has been shown to peak at 12 hours after RT in the SGZ, and by 48 hours postirradiation, the number of proliferating cells in the SGZ was reduced by 93–96% [128]. This data suggests that impairment of neurogenesis within the hippocampus, specifically within the SGZ/dentate gyrus, may be at least partly responsible for the cognitive impairments seen after brain irradiation [127].

At our own institution, we have completed a dosimetric feasibility study using helical tomotherapy (TomoTherapy, Madison, WI) to restrict dose to the hippocampus and the rest of the limbic circuit while simultaneously treating the rest of the brain to full dose using treatment schedules for both PCI and WBRT (30 Gy in 15 fractions and 35 Gy in 14 fractions, resp.) [129]. We found that we were able to reduce the mean dose to the hippocampus to 11.7 Gy and 14 Gy in the PCI and WBRT plans, respectively; this constitutes a mean relative reduction in BED Gy_2_ of 72.9% and 73.4% in the PCI and WBRT plans, assuming an alpha/beta ratio of 2 for late brain adverse effects [129].

Investigators in Vancouver have recently published the results of a hippocampal sparing feasibility study in which they successfully treated the whole brain to full dose (32.25 Gy) while simultaneously treating the bilateral hippocampi to a mean dose of less than 6 Gy and boosting the metastatic lesions to 63–70.8 Gy depending on diameter, using volumetric modulated arc therapy (VMAT) [130]. Their mean treatment time was only 3.6 minutes [130]. 

Reduction of radiation exposure of the hippocampus appears to be conceptually safe and dosimetrically feasible and represents one of the most promising strategies for maintaining the efficacy of WBRT and PCI, while minimizing the morbidity previously described.

## 7. Sparing of the Limbic Circuit

**Figure 2:**
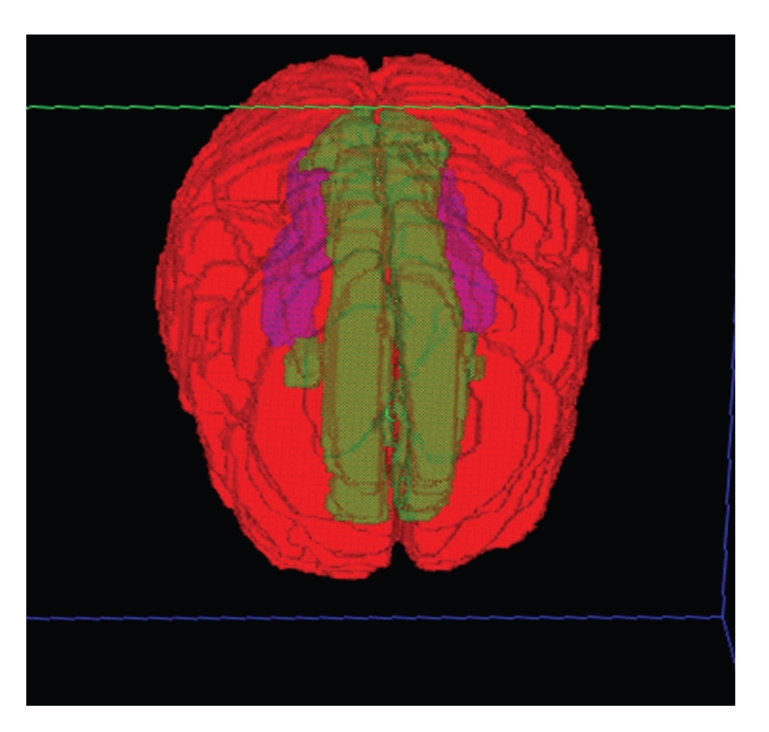
Axial 3D rendering of the hippocampus (purple) and limbic circuit (yellow) contours.

**Figure 3:**
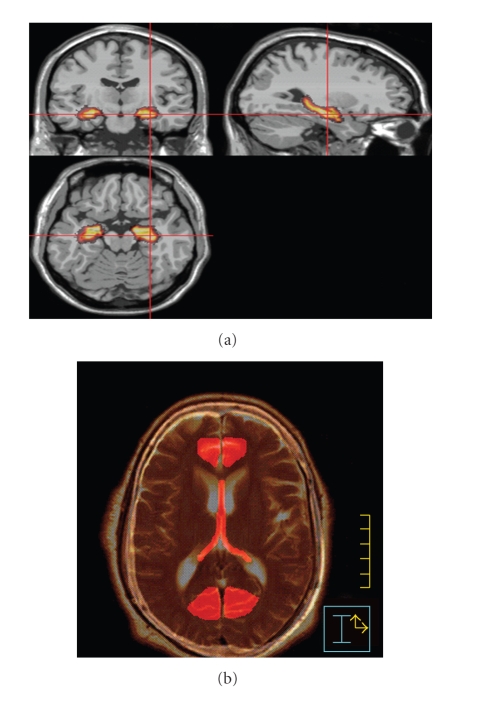
MRI images demonstrating location of (a) hippocampus, and (b) fornix and cingulate gyrus (Marsh et al. [121]). (a) Hippocampus contoured on coronal, sagittal, and axial MRI images. (b) Axial MRI demonstrating location of fornix and cingulated gyrus (anterior and posterior).

While dosimetric sparing of the hippocampus may reduce the loss of memory consolidation noted after cranial radiotherapy, selective sparing of the hippocampal formation (dentate gyrus and cornu ammonis) may not be sufficient. The hippocampal formation is only one of a number of structures which constitute the limbic circuit, or circuit of Papez [123]. The limbic circuit (Figures 1, 2, and 3) consists of 2 adjacent arches within the brain which bound the ventricular system [123]. The inner arch (amygdala, hippocampus [cornu ammonis and dentate gyrus], fornix, and mammillary bodies) is separated from the outer arch (parahippocampal gyrus, cingulum, cingulate gyrus, induseum griseum, and paraterminal gyrus) by the hippocampal sulcus and corpus callosum [123]. 

This circuit is critical to a number of vital brain functions: integration and consolidation of new memories, special orientation, emotional responses and behavior, autonomic responses to external stimuli, and fine motor coordination (among others) [123]. The two structures most intimately associated with the hippocampus include the parahippocampal gyrus and amygdaloid complex [123]. The parahippocampal gyrus is critical to memory encoding and retrieval of memories, and its ventral-most portion, called the entorhinal cortex, is the major source of afferent signals to the hippocampus [123]. The amygdaloid complex, or amygdala, is involved in memory modulation (required for long-term memory consolidation and the association of memory with emotional and physiological states) and emotional learning (fear reactions, imprinting, breeding behaviors, etc.) [123]. These three structures—the hippocampal formation, parahippocampal gyrus, and amygdaloid complex—form a functional unit within the medial temporal lobe, and true memory consolidation and learning require the function of all three structures [123].

Other structures that constitute the limbic circuit include the cingulate gyrus (which regulates autonomic responses to various stimuli and is involved in attention/concentration), cingulum (white matter bundle adjacent to the cingulate gyrus which connects the cingulated gyrus and prefrontal area to the parahippocampal gyrus), fornix and mammillary bodies (discussed previously) [123]. This circuit is directly connected to, and modulates the function of, a number of other critical intracranial structures including the hypothalamus, thalamus, prefrontal and orbitofrontal cortices, and nucleus accumbens (the brain's “pleasure center”) [123]. The function of the circuit as a whole is to process memory, support learning (cognitive, emotional, and autonomic), regulate emotional states, and assist in spatial orientation [123]. Interestingly, these are the most commonly reported neurocognitive deficits seen as components of late toxicity from cranial irradiation [10]. This suggests that it is damage to this critical circuit which is responsible for many of the late sequelae of therapy. Thus, dosimetric sparing of the limbic circuit may reduce such sequelae.

At our institution, we have performed a retrospective review of 697 intracranial metastases in 107 patients. Limbic metastases accounted for only 5.2% of all lesions. Among patients with oligometastatic disease (1–3 metastases), the rate was even lower at 4.8% [121]. The rate of hippocampal formation involvement was less than 1% among oligo-metastatic patients, while 3.9% of lesions involved the rest of the limbic circuit [121]. 

In a subsequent dosimetric feasibility study conducted in our department, we found that it was possible to restrict the mean dose to the limbic circuit to 15.1 Gy and 17.7 Gy in PCI (30 Gy in 15 fractions) and WBRT (35 Gy in 14 fractions) plans, respectively, using helical TomoTherapy [129]. This constitutes a mean reduction in BED Gy_2_ of 62.2% and 63.3% for the PCI and WBRT plans, respectively, assuming an alpha/beta ratio of 2 for late brain side effects [129]. The mean doses and reductions in BED Gy_2_ for the hippocampal formation (which was contoured separately) were even more pronounced, as discussed previously [129].

Reduction of radiation exposure for the limbic circuit may be safe, is dosimetrically feasible, and should reasonably be expected to reduce rates of memory loss in patients treated with cranial radiation therapy. These benefits may expand upon those obtained by dosimetric sparing of the hippocampal formation alone.

## 8. Neural Stem Cell Sparing

**Figure 4:**
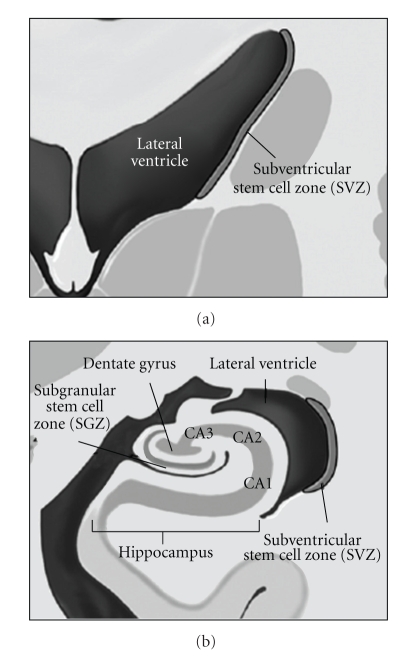
Neural stem cell (NSC) regions of the brain (Barani et al. [143]).

It is now known that the human brain contains regions of mitotically active cells which retain the ability to divide and differentiate along either neural or glial cell lines throughout life [124]. These stems, known as neural stem cells (NSC, Figure 4), are located in two specific areas of the brain: the subgranular zone (SGZ) within the dentate gyrus (part of the hippocampus) and the subventricular zone (SVZ) adjacent to the lateral aspect of the temporal horn and the occipital trigone region of the lateral ventricles [131, 132]. These cells are capable of increasing their mitotic rate under the influence of appropriate stimuli (e.g., brain trauma, stroke, radiation exposure, etc.) and can migrate through the brain to damaged areas and repopulate areas of cortical neuronal loss or white matter damage [133, 134]. They are also involved in replacing the neurons that are lost as a result of neurodegenerative disorders, and are important in learning [135–142]. 

It is hypothesized that the loss of these vital cells results in the inability to repair radiation-induced damage to normal brain tissue, the phenotypic expression of which is manifest as memory loss, loss of executive function, and the other late sequelae of therapy [144]. Preservation of the NSC compartments during the administration of WBRT or PCI should result in maintenance of the ability of the brain to repair the damage generated by cranial irradiation and help preserve neurocognitive function.

Barani et al. have shown that it possible to identify and dosimetrically reduce dose to these regions using intensity-modulated radiation therapy (IMRT) while treating a patient using treatment schedules applicable to whole brain radiotherapy and a primary high-grade glioma [143]. They selected a patient with a right paraventricular tumor and prepared two IMRT treatment plans for the patient; the first plan assumed that this mass represented a high-grade glioma and treated the patient to 60 Gy in 30 fractions, while the second plan assumed that this mass was a metastatic lesion and treated the patient with WBRT to 37.5 Gy in 15 fractions followed by a stereotactic radiosurgery (SRS) boost of 18 Gy. They were able to reduce the dose to the NSC compartment by 65% in the WBRT plan and 25% in the high-grade glioma plan, even in this patient with a tumor with an unfavorable location (adjacent to the right SVZ) [143].

Sparing of the NSC may be the single most effective method of mitigating the negative effects of WBRT if these cells survive and are able to repair radiation-induced damage. The critical role of radiation-induced damage to the NSC compartment as a cause of the cognitive dysfunction seen after cranial irradiationhas had been recently reviewed, and a considerable amount of evidence is available from in vitro and animal studies which exists in support of this hypothesis [145–151]. There is now also evidence from human studies of patients treated with radiotherapy for malignant brain tumors that cranial irradiation reduces the number of viable NSC [152].

Investigators at our institution have completed a dosimetric feasibility study in which patients are treated with either PCI (30 Gy in 15 fractions) or WBRT (35 Gy in 14 fractions) to full dose with simultaneous dosimetric sparing of the hippocampal formation (including the SGZ) and NSC compartment (SVZ, a 5 mm expansion around the lateral ventricle) [153]. The hippocampus and the rest of the NSC compartment received a mean dose of 11.5 Gy in the PCI plans and 11.8 Gy in the WBRT plans; this constitutes a 65.8% reduction in BED Gy_10_ for the NSC compartment in the PCI plans and a 70.8% reduction in the WBRT plans (assuming an *α*/*β* ratio of 10 for the NSC in the SGZ and SVZ) [153]. The corresponding reductions in BED Gy_2_ for the non-NSC component of the hippocampal formation were 73.8% and 78.6% in the PCI and WBRT plans, respectively (assuming an *α*/*β* ratio of 2 for the non-NSC/differentiated portion of the hippocampal formation) [153]. All NSC-preserving plans were generated using helical TomoTherapy [153]. Thus, dosimetric sparing of the NSC compartment has been proven feasible, and we believe that pilot studies employing NSC sparing IMRT would be appropriate.

## 9. Conclusions

While WBRT and PCI remain effective therapies for the treatment of, and prophylaxis against the development of, intracranial metastases, they are associated with both acute and late adverse effects. These adverse effects have spurred interest in either eliminating or reducing the use of these modalities or finding ways to reduce the incidence of adverse sequalae. The hippocampus and limbic circuit are also promising avoidance structures, as many of the established late adverse effects of both WBRT and PCI are neurocognitive deficits which result from damage to these structures. There is currently interest at a number of centers in selectively sparing these sensitive regions, while treating the remainder of the at risk brain to an effective dose. In the setting of WBRT for brain metastases, and in the setting of PCI, recent data suggests that sparing of these critical regions may not compromise intracranial control. A novel potential approach to reducing the late sequelae of cranial irradiation is selective dosimetric avoidance of the brain's neural stem cell (NSC) compartment, which would help maintain the brain's natural ability to repair damage created by radiation exposure.

 Clinical trials utilizing selective sparing of critical brain regions which prospectively incorporate neurocognitive testing are justified and may potentially lead to the modernization of a classical technique in radiation oncology. These trials will likely employ one or another form of intensity-modulated radiation therapy (IMRT), a technique which allows for steep dose gradients to be generated around even irregular or concave targets, as suggested by Movsas at the November 2009 ASTRO presentation of RTOG 0214 [24]. The dosimetric feasibility study by Gutiérrez et al. and Marsh et al. employed helical TomoTherapy, while Barani et al. utilized traditional inverse-planned IMRT [120, 129, 143, 153]. Investigators at our institution have recently opened a Phase II trial in which patients with limited stage SCLC (who demonstrate a complete response to treatment of their primary disease) and single resected brain metastases (with no evidence of metastatic disease outside the CNS) will be treated with limbic circuit-sparing PCI (30 Gy in 15 fractions) or WBRT (37.5 Gy in 15 fractions) using helical TomoTherapy [154]. Baseline and follow-up cognitive function will be assessed with a formal battery of neurocognitive tests, and results will be compared with historical controls. Interim safety data will be reported to document any failures within the spared regions of the brain.

## Figures and Tables

**Table 1:** RTOG RPA Classification for brain metastases.

Class	Characteristics	Median Survival (months)
I	KPS 70 or greater, age 65 years or less, primary disease controlled, no extracranial metastases	7.1

II	All others	4.2

III	KPS < 70	2.3

**Table 2:** RTOG/EORTC Late Morbidity Scoring System for Brain.

Grade 0	None

Grade 1	Mild headache, slight lethargy

Grade 2	Moderate headache, great lethargy

Grade 3	Severe headache, severe CNS dysfunction (partial loss of power or dyskinesia)

Grade 4	Seizure, paralysis, coma

Grade 5	Death

**Table 3:** NCI Common Toxicity Criteria Version 2.0 Summary.

Grade 0	Normal

Grade 1	Confusion/disorientation which resolves without sequelae, somnolence/dizziness/extrapyramidal symptoms/insomnia/memory loss/mood alterations/neuropathy/personality changes/pyramidal symptoms/tremor/vertigo not interfering with daily function, mild atrophy or limited T2 hyperintensities on MRI (<1/3 of cerebrum), nystagmus

Grade 2	Persistent confusion/disorientation/poor attention span not interfering with daily function, somnolence/dizziness/extrapyramidal symptoms/insomnia/memory loss/mood alterations/neuropathy/personality changes/pyramidal symptoms/tremor/vertigo/cranial neuropathies not interfering with activities of daily living (ADL), moderate atrophy or more extensive T2 hyperintensities on MRI (1/3-2/3 of cerebrum) extending into centrum ovale, nystagmus

Grade 3	Delusions, hallucinations, syncope, severe atrophy or near total T2 hyperintensities on MRI +/− focal white matter necrosis, persistent confusion/disorientation/poor attention span/somnolence/dizziness/extrapyramidal symptoms/insomnia/memory loss/mood alterations/neuropathy/personality changes/pyramidal symptoms/tremor/vertigo/cranial neuropathies interfering with activities of daily living (ADL)

Grade 4	Bedridden/disabled due to brain toxicity, requiring hospitalization doe to risk to self/others, psychotic, unable to communicate, amnesia, diffuse calcification or necrosis, paralysis

Grade 5	Death
